# Metabolic fingerprinting, antioxidant characterization, and enzyme-inhibitory response of *Monotheca buxifolia* (Falc.) A. DC. extracts

**DOI:** 10.1186/s12906-020-03093-1

**Published:** 2020-10-16

**Authors:** Joham Sarfraz Ali, Hammad Saleem, Abdul Mannan, Gokhan Zengin, Mohamad Fawzi Mahomoodally, Marcello Locatelli, Syafiq Asnawi Zainal Abidin, Nafees Ahemad, Muhammad Zia

**Affiliations:** 1 Department of Biotechnology, Quaid-i-Azam University Islamabad, Islamabad, 45320 Pakistan; 2 School of Pharmacy, Monash University, Jalan Lagoon Selatan, 47500 Bandar Sunway, Selangor Darul Ehsan Malaysia; 3 Institute of Pharmaceutical Sciences (IPS), University of Veterinary & Animal Sciences (UVAS), Lahore, Pakistan; 4 Department of Pharmacy, COMSATS University Abbottabad campus Abbottabad, Abbottabad, Pakistan; 5 Department of Biology, Faculty of Science, Selcuk University, Campus/Konya, Turkey; 6 Department of Health Sciences, Faculty of Science, University of Mauritius, Réduit, Mauritius; 7 Department of Pharmacy, University ‘G. d’Annunzio” of Chieti-Pescara, 66100 Chieti, Italy; 8 Liquid Chromatography Mass Spectrometery (LCMS) Platform, Monash University, Jalan Lagoon Selatan, 47500 Bandar Sunway, Selangor Darul Ehsan Malaysia

## Abstract

**Background:**

Ethnobotanical and plant-based products allow for the isolation of active constituents against a number of maladies. *Monotheca buxifolia* is used by local communities due to its digestive and laxative properties, as well as its ability to cure liver, kidney, and urinary diseases. There is a need to explore the biological activities and chemical constituents of this medicinal plant.

**Methods:**

In this work, the biochemical potential of *M. buxifolia* (Falc.) A. DC was explored and linked with its biological activities. Methanol and chloroform extracts from leaves and stems were investigated for total phenolic and flavonoid contents. Ultrahigh-performance liquid chromatography coupled with mass spectrometry (UHPLC–MS) was used to determine secondary-metabolite composition, while high-performance liquid chromatography coupled with photodiode array detection (HPLC–PDA) was used for polyphenolic quantification. In addition, we carried out in vitro assays to determine antioxidant potential and the enzyme-inhibitory response of *M. buxifolia* extracts.

**Results:**

Phenolics (91 mg gallic-acid equivalent (GAE)/g) and flavonoids (48.86 mg quercetin equivalent (QE)/g) exhibited their highest concentration in the methanol extract of stems and the chloroform extract of leaves, respectively. UHPLC–MS analysis identified a number of important phytochemicals, belonging to the flavonoid, phenolic, alkaloid, and terpenoid classes of secondary metabolites. The methanol extract of leaves contained a diosgenin derivative and polygalacin D, while kaempferol and robinin were most abundant in the chloroform extract. The methanol extract of stems contained a greater peak area for diosgenin and kaempferol, whereas this was true for lucidumol A and 3-*O*-*cis*-coumaroyl maslinic acid in the chloroform extract. Rutin, epicatechin, and catechin were the main phenolics identified by HPLC–PDA analysis. The methanol extract of stems exhibited significant 2,2-diphenyl-1-picrylhydrazyl (DPPH) and 2,2′-azino-bis(3-ethylbenzothiazoline-6-sulfonic acid (ABTS) radical-scavenging activities (145.18 and 279.04 mmol Trolox equivalent (TE)/g, respectively). The maximum cupric reducing antioxidant capacity (CUPRAC) (361.4 mg TE/g), ferric-reducing antioxidant power (FRAP) (247.19 mg TE/g), and total antioxidant potential (2.75 mmol TE/g) were depicted by the methanol extract of stems. The methanol extract of leaves exhibited stronger inhibition against acetylcholinesterase (AChE) and glucosidase, while the chloroform extract of stems was most active against butyrylcholinesterase (BChE) (4.27 mg galantamine equivalent (GALAE)/g). Similarly, the highest tyrosinase (140 mg kojic-acid equivalent (KAE)/g) and amylase (0.67 mmol acarbose equivalent (ACAE)/g) inhibition was observed for the methanol extract of stems.

**Conclusions:**

UHPLC–MS analysis and HPLC–PDA quantification identified a number of bioactive secondary metabolites of *M. buxifolia*, which may be responsible for its antioxidant potential and enzyme-inhibitory response. *M. buxifolia* can be further explored for the isolation of its active components to be used as a drug.

## Background

Ethnobotanical plants are considered the provenance of medicines possessing therapeutic potency to cure ailments and fight pathogenic maladies. Distant sanctioned systems of medicine, such as Ayurvedic, Chinese, and Unani medicine, utilize medicinal plants having remedial properties. The concomitant amelioration in technology and science has amplified the global use of medicinal plants due to their pharmacological and nutraceutical potential, highlighting their antioxidant, antimicrobial, anticancer, and enzyme-inhibitory properties [1]. Plants and/or plant-based products allow for the isolation of active components against a number of maladies [2]. Such compounds also function as preeminent agents, playing a key role in neutralizing or scavenging free radicals and decomposing peroxides, amongst others. The standardization of plant-based remedies assures the quality and international acceptability of miraculous agents, and much effort is demanded in this respect [3].

Pakistan has diverse genetic resources and a rich floristic wealth of medicinal plants due to favorable climatic conditions. Secondary metabolites are profiled using different techniques, which are then linked with biological activities, before further isolation of the active ingredients in an effort to cure diseases. *Monotheca buxifolia* belongs to the family Sapotaceae that comprises 800 species and 65 genera. Gurguri is the local name of its fresh fruit that is sold in markets due to its ethnobotanical significance [4]. *M. buxifolia* is found in barren hilly areas [5]. In Pakistan, it is widely present in Balochistan (i.e., Zhob, Gorakh Hills, and Loralai) and Khyber Pakhtunkhwa (Kohat, Drosh, Chitral, and Attock Districts). Its distribution is also observed in tribal areas along the border of Afghanistan, i.e., Mohmand Agency and Darraadamkhel [4]. The plant is used as folk medicine in South Asia (Pakistan, India, and Afghanistan) and the Middle East, Iran, and Iraq. According to folk knowledge, *M. buxifolia* has digestive and laxative properties, and the leaves are used to treat liver, kidney, and urinary diseases [6]. The fruit extract has hepatoprotective, urease-inhibitory, and antibacterial activities [7–9], and it is also known for its pain-, inflammation-, and pyrexia-ameliorating properties, mainly due to its oleanolic-acid and isoquercetin contents [10]. A previous study showed that the *M. buxifolia* fruit has a high amount of phenolics and flavonoids, and it depicts free-radical-scavenging activity [11]. The leaves of *M. buxifolia* are chemically enriched with flavonoids, terpenoids, saponins, anthraquinones, cardiac glycosides, tannins, and reducing sugars depicting high antioxidant activity [12]. The fruit also possesses significant antibacterial and cytotoxic properties, and it is enriched with lupeol and α-amyrin [13].

Keeping in view the medicinal value of this plant, the present study was designed to evaluate the phytochemical composition (phenolic and flavonoid contents via ultrahigh-performance liquid chromatography coupled with mass spectrometry (UHPLC–MS) analysis, and high-performance liquid chromatography coupled with photodiode array detection (HPLC–PDA)) and biological potential (including the antioxidant—2,2-diphenyl-1-picrylhydrazyl (DPPH), 2,2′-azino-bis(3-ethylbenzothiazoline-6-sulfonic acid (ABTS), ferric-reducing antioxidant power (FRAP), cupric reducing antioxidant capacity (CUPRAC), phosphomolybdenum, and metal chelation—and enzyme-inhibitory—acetylcholinesterase (AChE), butyrylcholinesterase (BChE), amylase, glucosidase, and tyrosinase—properties) of *M. buxifolia* leaf and stem extracts. In addition, principal component analysis (PCA) statistical studies were carried out to explain the association of bioactive contents with biological activities. These results will provide guidelines for isolating the active constituents from this plant to be employed in pharmaceutical studies.

## Methods

### Collection and extraction of plant material

**Fig. 1:**
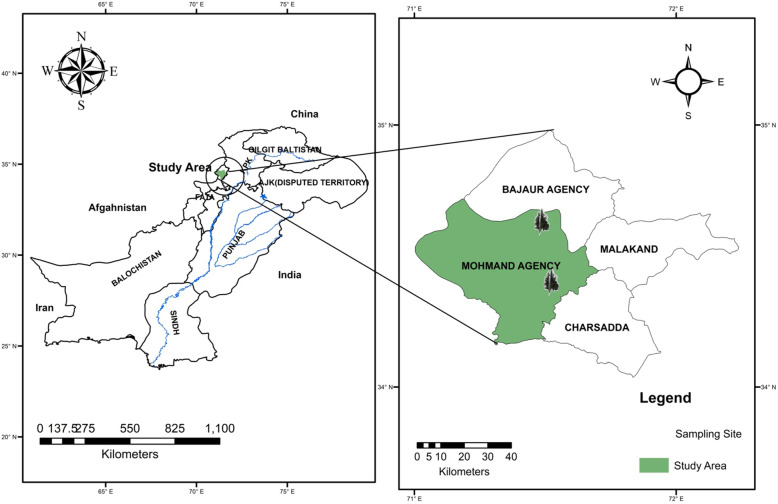
Map of Pakistan showing *Monotheca buxifolia* plant collection site Mohmand Agency

Fresh plant material (leaves and stems) of *Monotheca buxifolia* was collected in June 2016 from the Mohmand Agency Mountain, Khyber Pakhtunkhwa, Pakistan (Fig. 1). The plant was identified by Dr. Rizwana Aleem Qureshi (Taxonomist Department of Plant Sciences, Quaid-e-Azam University, Islamabad, Pakistan), and a herbarium sample was deposited (voucher ID BIT-4220, Herbarium Quaid-i-Azam University, Islamabad, Pakistan). The plant material was washed, dried under shade, and powdered using an electric grinder. The powdered material (leaves and stems) was separately macerated with analytical-grade methanol (1:3) and subjected to sonication for 30 min in an ultrasonic bath at room temperature [14]. The marc was filtered through a muslin cloth, followed by filtration through Whatmann filter paper No. 1. The residue was again dipped in methanol, and this procedure was repeated thrice. The filtrates were combined and concentrated using a rotary evaporator (Rotovapor R 200 Buchi, Flawil Switzerland) at 40 °C. The residue was macerated in chloroform (1:3), and the same procedure was followed as stated above to obtain the chloroform extract. The extracts were stored at 4 °C until further use.

### Determination of Total phenolic and flavonoid contents

The total phenolic and flavonoid contents were evaluated in the extracts following a previously reported protocol [15]. Total phenolic constituents are reported as gallic-acid equivalent (mg GAE/g extract), while total flavonoid contents are reported as quercetin equivalent (mg QE/g extract).

### Phytochemical composition of extracts

The secondary-metabolite profiling of methanol and chloroform extracts of *M. buxifolia* leaves and stems was assessed by utilizing standard reverse-phase (RP)-UHPLC–MS analysis as described previously [16]. Similarly, 22 polyphenolic components (gallic acid, catechin, chlorogenic acid, *p*-OH benzoic acid, vanillic acid, epicatechin, syringic acid, 3-OH benzoic acid, 3-OH-4-MeO benzaldehyde, *p*-coumaric acid, rutin, sinapinic acid, *t*-ferullic acid, naringin, 2.3-diMeO benzoic acid, benzoic acid, *o*-coumaric acid, quercetin, harpagoside, *t*-cinnamic acid, naringenin, and carvacrol; standards purchased from Sigma Aldrich Milan, Italy) were also determined in extracts using HPLC–PDA analysis following a previously reported protocol [15].

### Antioxidant assays

Previously reported standard in vitro methods [17] were followed to estimate the antioxidant properties of all the extracts, including their free-radical-scavenging property (DPPH and ABTS), reducing power potential (FRAP and CUPRAC), phosphomolybdenum-based total antioxidant capacity, and metal-chelating ability. The outcomes are presented as Trolox equivalents in all assays except for the metal-chelating assay where ethylenediaminetetraacetic acid (EDTA) was used as a standard.

### Enzyme-inhibitory assays

The enzyme-inhibitory capacity of all the concentrates against acetylcholinesterase (AChE), butyrylcholinesterase (BChE), tyrosinase, α-amylase, and α-glucosidase was explored following previously reported standard in vitro bioassays [17]. Galantamine was used as a reference for AChE and BChE, and the cholinesterase-inhibitory potential was estimated as mg galantamine equivalent (GALAE)/g extract. The α-amylase- and α-glucosidase-inhibitory potentials are presented as mmol acarbose equivalent (ACAE)/g extract, while the tyrosinase-inhibitory potential was recorded as mg kojic-acid equivalent (KAE)/g extract.

### Statistical analysis

All assays were carried out in triplicate. The results are expressed as mean values ± standard deviation (SD). The activities of extracts were differentiated through one-way analysis of variance (ANOVA), followed by Tukey’s honestly significant difference post hoc test with α = 0.05. The analysis was carried out using SPSS v. 14.0. Principal component analysis (PCA) using XLSTAT was applied to the resultant variables from the phytochemical analysis and biological assays.

## Results

### Phytochemical composition of extracts

Determination of the free-radical-scavenging activity, total reducing power, and total antioxidant capacity, and phytochemical (total phenolics and flavonoids) analyses of methanol and chloroform extracts of leaves and stems of *Monotheca buxifolia* were performed. Phytochemical analysis was also extended using UHPLC–MS and HPLC–PDA for untargeted and targeted metabolites. Clinically important enzyme-inhibitory assays were also performed. Table 1 shows that the methanol and chloroform extracts of *M. buxifolia* leaves and stems contained a significant amount of phenolics and flavonoids. The amount of total phenolic content was higher in the methanol extract of leaves (69.84 mg GAE/g extract) and stems (91.00 mg GAE/g extract) as compared to the chloroform extract. The leaves had a significant amount of flavonoids in both methanol (40.11 mg RE/g) and chloroform (48.86 mg RE/g) extracts.

**Table 1:** Phytochemical constituents and antioxidant activities of *M. buxifolia* leaves and stem extracts

Extract		Phytochemical contents		Radical Scavenging Activity		Reducing Power Assays		Ferrous ion Chelation	Total Antioxidant Capacity
		Total phenolic content (mgGAE/)	Total flavonoid content (mgRE/g)	DPPH (mmolTE/g)	ABTS (mmol TE/g)	CUPRAC (mgTE/g)	FRAP (mgTE/g)	Metal chelating (mgEDTAE/g)	Phosphomolybdenum (mmolTE/g)
**Leaves**	Methanol	69.84 ± 1.87^c^	40.11 ± 0.94^b^	132.14 ± 0.68^c^	257.25 ± 4.04^c^	253.96 ± 5.69^c^	197.36 ± 8.05^b^	6.24 ± 0.13^c^	2.10 ± 0.12^b^
	Chloroform	50.21 ± 0.87^d^	48.86 ± 0.75^a^	81.62 ± 4.25^d^	160.96 ± 4.29^d^	182.67 ± 1.85^d^	121.52 ± 4.94^c^	15.95 ± 1.03^a^	2.29 ± 0.22^b^
**Stem**	Methanol	91.00 ± 2.24^a^	7.37 ± 0.22^c^	145.18 ± 0.25^a^	279.04 ± 0.52^a^	361.40 ± 11.02^a^	247.19 ± 8.49^a^	2.90 ± 0.46^d^	2.75 ± 0.16^a^
	Chloroform	73.98 ± 2.22^b^	7.68 ± 0.16^c^	138.51 ± 0.94^b^	271.37 ± 0.35^b^	267.96 ± 10.33^b^	197.57 ± 9.15^b^	8.88 ± 0.83^b^	2.50 ± 0.23^ab^

All values expressed are means ± S.D. of three parallel measurements. GAE: Gallic acid equivalents; RE: Rutin equivalents; TE: trolox equivalent; EDTAE: EDTA equivalent. Different letters within column indicate significant differences in the extracts (*p* < 0.05)

**Fig. 2:**
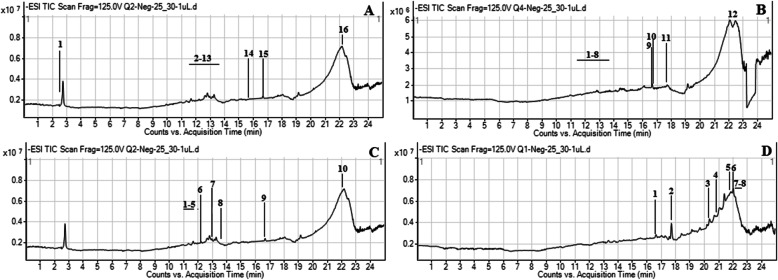
UHPLC-MS total ion chromatograms (TICs) of *M. buxifolia* leaves methanol extract (**a**); *M. buxifolia* leaves chloroform extract (**b**); *M. buxifolia* stem methanol extract (**c**); (*M. buxifolia* stem chloroform extract (**d**)

The secondary-metabolite components of *M. buxifolia* leaf and stem extracts were determined using liquid chromatography coupled with mass spectrometry. A typical chromatogram of the extracts with mass-spectrometric detection in negative ion mode exhibited complex patterns of peaks (Fig. 2a–d). UHPLC–MS analysis of the methanol extract of leaves revealed the presence of 16 compounds (Table 2), the majority of which were flavonoids, phenolics, and terpenoid derivatives. The most abundant components identified were a diosgenin derivative (an alkaloid) and polygalacin D (an organooxygen). Flavonoids such as kaempferol derivatives and robinin acquired major peaks in the UHPLC–MS analysis of the chloroform extract of leaves. UHPLC–MS analysis of the methanol extract of stems revealed the presence of 10 different compounds (Table 3). The alkaloid diosgenin had the greatest peak area (1353.63), followed by the flavonoid kaempferol. Moreover, the chloroform extract of stems depicted the presence of eight compounds (Table 3), including lucidumol A, 3-*O*-*cis*-coumaroyl maslinic acid (a terpenoid derivative), and mangostenone B (a benzopyran), as the major components. The results also suggested the occurrence of other important metabolites, the majority of which belonged to diverse classes including flavonoids, phenolics, terpenoids, alkaloids, and fatty-acid derivatives. HPLC–PDA analysis was carried out to gain insight into the polyphenolic profile of *M. buxifolia* leaf and stem extracts (Table 4). The results show that the methanol extract of leaves and the chloroform extract of stems contained the most phenolic compounds. Epicatechin was detected in all samples with maximum quantification (1.29 μg/mg) in the methanol extract of stems. Rutin and catechin were also present in all samples except for the chloroform extract of stems and leaves. However, catechin was quantified in a higher amount in the methanol extract of stems as compared to the other extracts. Similarly, rutin was quantified in a significantly higher amount in the chloroform extract of leaves as compared to the other extracts.

**Table 2:** UHPLC-MS of *M. buxifolia* leave extracts (negative ionization mode)

S/No	RT (min)	B. Peak (m/z)	Compound name	Comp. class	Mol. formula	Mol. Mass
***M. buxifolia*** **Leaves Methanol extract**						
**1**	2.574	225.06	Glucoheptonic acid	Sugar Acid	C_7_ H_14_ O_8_	226.06
**2**	11.558	739.20	Robinin	Flavonoid	C_33_ H_40_ O_19_	740.21
**3**	11.559	771.19	Kaempferol 3-glucoside-7-sophoroside	Flavonoid	C_33_ H_40_ O_21_	772.20
**4**	11.761	479.08	3,5,7,2′,3′,4′-Hexa hydroxy flavone 3-glucoside	Flavonoid	C_21_ H_20_ O_13_	480.09
**5**	11.85	755.20	Kaempferol 3-(2G-glucosylrutinoside)	Flavonoid	C_33_ H_40_ O_20_	756.21
**6**	11.889	609.14	Robinetin 3-rutinoside	Phenolic	C_27_ H_30_ O_16_	610.15
**7**	12.109	449.07	Myricetin 3-alpha-L-arabinopyranoside	Flavonoid	C_20_ H_18_ O_12_	450.08
**8**	12.214	463.08	8-Hydroxyluteolin 8-glucoside	Flavonoid	C_21_ H_20_ O_12_	464.096
**9**	12.582	433.07	Avicularin	Flavonoid	C_20_ H_18_ O_11_	434.08
**10**	12.962	1353.63	Diosgenin 3-[glucosyl-(1- > 4)-[glucopyranosyl-(1- > 6)]-glucopyranosyl-(1- > 4)-rhamnosyl-(1- > 4)-[rhamnosyl-(1- > 2)]-glucoside]	Alkaloid	C_63_ H_102_ O_31_	1354.63
**11**	13.057	171.06	cis-4-octenedioic acid	Fatty Acid	C_8_ H_12_ O_4_	172.07
**12**	13.359	1207.57	Polygalacin D	Organooxygens	C_57_ H_92_ O_27_	1208.58
**13**	13.699	269.10	Idebenone Metabolite (Benzenebutanoic acid, 2,5-dihydroxy-3,4-dimethoxy-6-methyl-)	Phenolic	C_13_ H_18_ O_6_	270.11
**14**	15.734	225.15	Dihydrojasmonic Acid, Methyl Ester	Phenolic	C_13_ H_22_ O_3_	226.15
**15**	16.667	221.11	(6S)-dehydrovomifoliol	Terpenoid	C_13_ H_18_ O_3_	222.12
**16**	22.201	471.34	Lucidumol A	Triterpenoid	C_30_ H_48_ O_4_	472.35
***M. buxifolia*** **Leaves Chloroform extract**						
**1**	11.558	739.20	Robinin	Flavonoid	C_33_ H_40_ O_19_	740.21
**2**	11.559	771.19	Kaempferol 3-glucoside-7-sophoroside	Flavonoid	C_33_ H_40_ O_21_	772.20
**3**	11.61	449.10	8-C-Glucopyranosyleriodictylol	Flavonoid	C_21_ H_22_ O_11_	243.05
**4**	11.85	755.20	Kaempferol 3-(2G-glucosylrutinoside)	Flavonoid	C_33_ H_40_ O_20_	756.21
**5**	12.109	449.07	Myricetin 3-alpha-L-arabinopyranoside	Flavonoid	C_20_ H_18_ O_12_	450.08
**6**	12.216	463.08	8-Hydroxyluteolin 8-glucoside	Flavonoid	C_21_ H_20_ O_12_	567.16
**7**	13.695	269.10	Idebenone Metabolite (Benzenebutanoic acid, 2,5-dihydroxy-3,4 dimethoxy-6-methyl-)	Phenolic	C_13_ H_18_ O_6_	270.11
**8**	15.73	225.14	Dihydrojasmonic Acid, Methyl Ester	Phenolics	C_13_ H_22_ O_3_	226.15
**9**	16.664	267.12	Kamahine C	Ketals	C_14_ H_20_ O_5_	268.13
**10**	16.666	221.11	(6S)-dehydrovomifoliol	Terpenoid	C_13_ H_18_ O_3_	222.12
**11**	17.713	356.17	Uplandicine	Alkaloid	C_17_ H_27_ N O_7_	357.17
**12**	22.205	471.34	Lucidumol A	Triterpenoid	C_30_ H_48_ O_4_	472.35

*RT* retention time, *B. peak* base peak

**Table 3:** UHPLC-MS of *M. buxifolia* stem extracts (negative ionization mode)

S/No	RT (min)	B.Peak (m/z)	Compound name	Comp. class	Mol. formula	Mol. Mass
***M. buxifolia*** **stem methanol extract**						
**1**	11.128	391.12	Shanzhiside	Iridoid glycoside	C_16_ H_24_ O_11_	527.09
**2**	11.317	577.13	Apigenin 7-(2″-E-p-coumaroylglucoside)	Flavonoid	C_30_ H_26_ O_12_	145.07
**3**	11.341	755.20	Kaempferol 3-(2G-glucosylrutinoside)	Flavonoid	C_33_ H_40_ O_20_	641.13
**4**	11.61	449.10	8-C-Glucopyranosyleriodictylol	Flavonoid	C_21_ H_22_ O_11_	243.05
**5**	11.672	581.22	(7’R)-(+)-Lyoniresinol 9′-glucoside	Lignan Glycoside	C_28_ H_38_ O_13_	244.90
**6**	12.216	463.08	8-Hydroxyluteolin 8-glucoside	Flavonoid	C_21_ H_20_ O_12_	567.16
**7**	12.952	1353.63	Diosgenin 3-[glucosyl-(1- > 4)-[glucopyranosyl-(1- > 6)]-glucopyranosyl-(1- > 4)-rhamnosyl-(1- > 4)-[rhamnosyl-(1- > 2)]-glucoside]	Alkaloid	C_63_ H_102_ O31	1354.63
**8**	13.695	269.10	Idebenone Metabolite (Benzenebutanoic acid, 2,5-dihydroxy-3,4-dimethoxy-6-methyl-)	Phenolic	C_13_ H_18_ O_6_	392.13
**9**	16.669	221.11	(6S)-dehydrovomifoliol	Terpenoid	C_13_ H_18_ O_3_	159.98
**10**	21.036	471.34	Lucidumol A	Triterpenoid	C_30_ H_48_ O_4_	353.07
***M. buxifolia*** **stem chloroform extract**						
**1**	16.63	221.11	(6S)-dehydrovomifoliol	Terpenoid	C_29_ H_42_ O_5_	222.12
**2**	17.714	356.17	Uplandicine	Alkaloid	C_30_ H_48_ O_5_	357.17
**3**	20.376	257.15	Cicutoxin	Fatty Alcohol	C_29_ H_46_ O_4_	258.16
**4**	20.816	461.19	Mangostenone B	Benzopyran	C_20_ H_24_ O_2_	462.20
**5**	21.814	297.24	cis-9,10-Epoxystearic acid	Lineolic Acid	C_16_ H_32_ N_6_ O_5_	298.25
**6**	22.066	339.23	Plastoquinone 3	Isoprenoid quinone	C_23_ H_32_ O_2_	340.24
**7**	22.164	471.34	Lucidumol A	Triterpenoid	C_30_ H_48_ O_4_	472.35
**8**	22.2	617.38	3-O-cis-Coumaroyl maslinic acid	Triterpenoid	C_39_ H_54_ O_6_	618.39

*RT* retention time, *B. peak* base peak

**Table 4:** HPLC-PDA polyphenolic quantification of the tested *M. buxifolia* leaves and stem extracts (μg/mg)

Polyphenoilic compounds	Leaves		Stem	
	Methanol	Chloroform	Methanol	Chloroform
Gallic acid	nd	Nd	nd	BLQ
Catechin	4.29 ± 0.52	Nd	7.75 ± 0.84	3.95 ± 0.86
Vanillic acid	nd	BLQ	nd	nd
Epicatechin	0.59 ± 0.05	0.89 ± 0.08	1.29 ± 0.95	0.82 ± 0.08
3-OH benzoic acid	nd	Nd	nd	0.79 ± 0.09
Rutin	0.74 ± 0.08	13.7 ± 1.54	0.45 ± 0.04	nd
Naringin	0.27 ± 0.03	Nd	nd	nd

Values are means SD of three measurements; BLD: below limit of detection < 0.1 μg/mL; BLQ: below limit of quantification < 0.2 μg/mL; nd: not detected

Chlorogenic acid, p-OH benzoic acid, Syringic acid, 3-OH-4-MeO benzaldehyde, p-coumaric acid, Sinapinic acid, t-ferullic acid, 2.3-diMeO benzoic acid, Benzoic acid, o-coumaric acid, Quercetin, Harpagoside, t-cinnamic acid, Naringenin, and Carvacrol were not detected in any of the tested extracts

### Antioxidant evaluation

Several different assays were performed including radical-scavenging (DPPH and ABTS), reducing power (CUPRAC and FRAP), ferrous-ion chelation, and phosphomolybdenum assays (Table 1). The methanol extract of stems showed prominent free-radical-scavenging activity and reducing power. The DPPH and ABTS assays presented values of 145.18 mmol TE/g and 279.04 mmol TE/g, respectively, for the methanol extract of stems (Table 1). The chloroform extract of leaves showed significant (15.95 mg EDTA equivalent (EDTAE)/g) ferrous-ion-chelating activity. All four extracts showed a nonsignificant difference for the phosphomolybdenum-based total antioxidant response.

### Enzyme-inhibitory assays

The enzyme-inhibitory capabilities of *M. buxifolia* extracts were assessed for five clinically important enzymes, which showed their significant potential (Table 5). The methanol extracts of both parts of the plant and the chloroform extract of stems were most active against AChE (4.70, 4.66, and 4.62 mg GALAE/g, respectively), while, for BChE, the chloroform extract of stems was most potent (4.27 mg GALAE/g). The stem extracts showed significant tyrosinase- (140.16 and 137.93 mg KAE/g inhibition by methanol and chloroform extracts, respectively) and amylase-inhibitory activities; however, all four extracts presented equal inhibition of glucosidase activity.

**Table 5:** Enzyme inhibition assays of *M. buxifolia* leaves and stem extracts

Extract		AChE (mgGALAE/g)	BChE (mgGALAE/g)	Tyrosinase (mgKAE/g)	Amylase (mmolACAE/g)	Glucosidase (mmolACAE/g)
Leaves	Methanol	4.70 ± 0.08^a^	3.01 ± 0.60^b^	132.90 ± 1.68^b^	0.56 ± 0.06^b^	59.50 ± 0.25^a^
	Chloroform	3.87 ± 0.07^b^	2.05 ± 0.20^c^	121.31 ± 0.38^c^	0.56 ± 0.02^b^	58.28 ± 0.37^a^
Stem	Methanol	4.66 ± 0.04^a^	3.95 ± 0.42^ab^	140.16 ± 2.27^a^	0.67 ± 0.03^a^	59.48 ± 0.10^a^
	Chloroform	4.62 ± 0.03^a^	4.27 ± 0.55^a^	137.93 ± 0.87^a^	0.66 ± 0.05^a^	59.09 ± 0.09^a^

All values expressed are means ± S.D. of three parallel measurements. GALAE: galatamine equivalent; KAE: kojic acid equivalent; ACAE: acarbose equivalent. Different letters within column indicate significant differences in the extracts (*p* < 0.05)

### PCA statistical evaluation

**Fig. 3:**
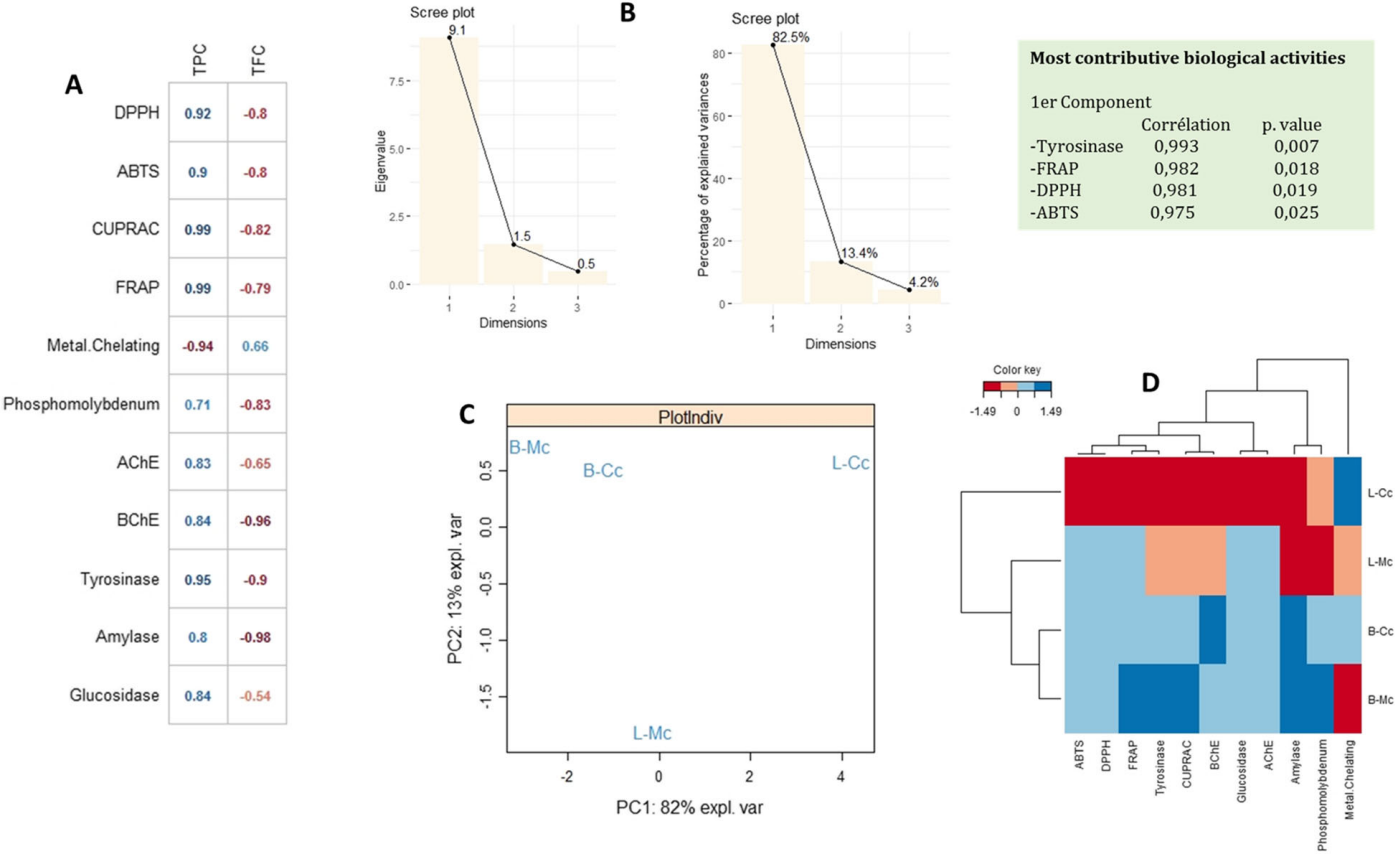
Statistical evaluations, **a**: Correlation coefficients between phyto constituents and biological activities ((r), *p < 0.05*); **b**: Eigen values and percentage of variability expressed by the factors; **c**: Projection of samples into the subspace PC1vsPC2; **d**: Heat map of extracts in according to bioactive compounds and biological activities

To assess the similarity of the biological activities expressed by the extracts and to analyze the correlations between variables, a principal component analysis (PCA) was performed. The Pearson correlation, as depicted in Fig. 3a, suggests a firm positive correlation between total phenolic content (*r* = 0.99–0.71) and antioxidant potential (except for metal chelation). However, the negative relationship between total phenolic content (*r* = − 0.94) and metal chelation may be due to the antagonistic or synergetic effect of phytochemicals or the presence of some nonphenolic chelators. Similarly, a strong positive relationship (*r* = 0.95–0.8) was recorded between phenolic content and enzyme-inhibitory activity. In contrast, in the case of flavonoid content, a strong negative correlation was noted with both antioxidant potential (*r* = − 0.8 to − 0.79) and enzyme-inhibitory activity (*r* = − 0.99 to − 0.54), except for metal-chelation activity, with which flavonoid content was positively correlated (*r* = 0.66). Overall, it was observed that the tyrosinase-inhibitory, FRAP, DPPH, and ABTS activities were the most contributory biological activities to the formation of the first principal component with *p-*values of 0.007, 0.018, 0.019, and 0.025, respectively. The bioactive compounds and the biological activity showed an 82.5% eigenvalue (Fig. 3b), in accordance with the strong correlation between the various activities and the extract components determined through HPLC–PDA.

## Discussion

In recent years, the biological activities (such as anticancer, antimicrobial, antioxidant, and anti-inflammatory) of phenolic compounds and their flavonoid subclass have captured significant attention due to their unique structural and functional properties [18]. Therefore, the determination of total phenolics and flavonoids in plant extracts is advantageous when linking their content with biological activities, which can be further exploited for medicinal use and an immune-boosting response. Table 1 shows that the methanol and chloroform extracts of *Monotheca buxifolia* leaves and stems contained a significant amount of phenolics and flavonoids. The total phenolic content was higher in the methanol extract as compared to the chloroform extract. Methanol dissolves the polar constituents of plants, including phenolics [19, 20]; therefore, it is considered the best solvent for the extraction of phenolic compounds. In the case of flavonoid content, the leaf extracts contained a higher amount of flavonoids as compared to the stem extracts.

UHPLC–MS analysis of the methanol extract of leaves revealed the presence of flavonoids, phenolics, and terpenoid derivatives, with the most abundant compounds being a diosgenin derivative and polygalacin D, whereas kaempferol and robinin were abundantly present in the chloroform extract of leaves. The UHPLC–MS analysis of the stem extract revealed the presence of diverse components including alkaloids, flavonoids, terpenoids, and benzopyran derivatives as the major components. The presence of diverse phytochemicals is in agreement with a previous study that reported a number of secondary metabolites from this plant [12]. Chromatographic fingerprinting, including HPLC- and mass-spectrum-based identification of compounds, is a powerful tool for the separation and recognition of phytoconstituents [21]. A number of food and medicinal plants have been processed for primary- and secondary-metabolite determination [22] to establish a possible link between their phytocomponents and beneficial bioactivities. It is well known that the polyphenolics and flavonoids, as well as their glycosides, are responsible for antioxidant, anticancer, and cardioprotective activity [23]. The HPLC–PDA analysis results showed that the leaf and stem extracts contained diverse phenolic compounds, with epicatechin, rutin, and catechin as the most abundant. Plants contain different classes of phenols, including polyphenols, flavonoids, phenolic acids, stilbenes, and lignins, which are involved in the protection against and cure of diseases such as cancers, cardiovascular diseases, diabetes, and others [23, 24]. Flavonoids are the most abundant polyphenol in human diets. Dietary polyphenols may activate endogenous defense systems and regulate cellular-signaling processes [25, 26]. Phenolic contents are also recognized to reduce the risk of chronic diseases, increase healthy lifespan, and promote active healthy aging [27]. For example, epicatechin and catechin have diverse biological properties, including antioxidant, antimicrobial, anti-inflammatory, antitumor, and cardioprotective activity [28, 29]. Rutin was also observed in a significant amount in the leaves and stems of *M. buxifolia*. Rutin has antioxidant properties, which enable protection from cellular damage caused by free radicals. It also helps to eliminate cholesterol from the body and maintain healthy collagen, and it has anti-inflammatory and anticarcinogenic properties, amongst others [30].

Antioxidants are agents that scavenge free radicals, preventing cellular damage [31]. Reductants are involved in donating hydrogen atoms and breaking down free-radical chains [32]. Phenols and reductants are good electron donors [33]. An inadequate supply of free radicals may damage DNA, protein, and lipid molecules, and it may disrupt membrane phospholipids, thereby leading to diseases and cancer [34]. The methanol extract of stems showed prominent DPPH and ABTS free-radical scavenging activity and reducing power. However, the chloroform extract significantly quenched ferrous ions, showing noteworthy chelating activity. Oxidative stress is related to many health issues such as malignancy, diabetes, cardiac and neurodegenerative disorders, amongst others. Synthetic antioxidants can be used to cure the diseases; however, they may damage vital organs. Therefore, natural antioxidants are of prime importance for better health [35]. The antioxidative response of *M. buxifolia*, such as its free-radical-scavenging activity, reducing power, and metal-chelating ability, was found to be linked to the presence of antioxidants [4, 14]. Furthermore, it was reported that the solvent used, the plant part used, the mode of processing, and the fractionation scheme influence the extraction of antioxidants and their activities. A number of phytochemicals, such as 3-OH benzoic acid and epicatechin, were reported to have antioxidative potential [36]. Chelation agents that bind to pro-oxidant metals are regarded as active secondary antioxidants [37, 38]. These results are also in agreement with studies showing that the antioxidant activity is proportional to the phenolic compound content, which is associated with the solvent used [39, 40]. The extracts in this study also presented antioxidant activity via the iron-chelation and radical-scavenging assays, thereby indicating the presence of both primary and secondary antioxidants. Primary antioxidants neutralize free radicals to prevent the initiation and propagation of oxidative chain reactions, while secondary antioxidants suppress radical formation and protect against oxidative damage [41].

Enzyme-inhibitory assays are a potent tool to assess the significant health benefits of medicinal plants, dietary supplements, and nutraceuticals [42]. Due to the drastic prevalence of several ailments, there is an urgency to address these health hazards, which commonly include Alzheimer’s disease and diabetes. *M. buxifolia* extracts showed prominent inhibitory activity against clinically important enzymes. The methanol extracts of both parts of the plant and the chloroform extract of stems were most active against AChE, while the chloroform extract of stems was the most potent against BChE. Acetylcholinesterase (AChE) is localized at the cholinergic synapses and regulates neurotransmission through rapid hydrolysis of the neurotransmitter acetylcholine into choline and acetate [43, 44]. Inhibition of AChE can increase the levels of acetylcholine, thereby providing symptomatic relief [45]. On the other hand, butyrylcholinesterase (BChE) is a serine hydrolase associates with lipid metabolism and indicators of metabolic syndrome such as body mass index, waist–hip ratio, waist circumference, weight, cholesterol, and triglyceride levels [46–48]. Increased BChE activity may also lead to a greater degradation of acetylcholine, which lowers the inhibitory effect on the production of cytokines [49]. Furthermore, amylase and glucosidase play a vital role in the digestion of carbohydrates. In diabetes and obesity, inhibition of these enzymes is essential to reduce carbohydrate digestion [50]. The stem extracts showed significant tyrosinase- and amylase-inhibitory activity; however, all fours extracts depicted equal activity inhibition of glucosidase activity. The significant enzyme-inhibitory potential of *M. buxifolia* extracts is therapeutically important, which suggests its potential use as promising antidiabetic medicine. Inhibition of α-amylase is an efficacious approach to overcoming postprandial hyperglycemia [51, 52]. Tyrosinase is one of the key enzymes responsible for the biosynthesis of melanin, and its inhibition is considered the best strategy to treat epidermal hyperpigmentation problems [53]. Tyrosinase also causes browning of fruits and vegetables, leading to quicker degradation and deterioration of nutritional value [54]. The use of tyrosinase inhibitors has attracted great attention in the cosmetic and pharmaceutical industries due to their preventive effects in pigmentation disorders. This scenario demands deliberate action involving the use of enzyme-inhibitory compounds as an effective strategy [54].

PCA analysis suggested a firm positive correlation between total phenolic content and antioxidant activity. This positive association of phenolics with antioxidant activity is in accordance with previous studies showing such a trend [42]. However, the negative relationship in a few cases may be due to the antagonistic or synergetic effect of phytochemicals or the presence of some nonphenolic chelators [17]. A strong correlation was also identified between enzyme-inhibitory potential and phytochemical content through biological assays and HPLC analysis. The bioactive compounds and the biological activity showed a considerable eigenvalue, in accordance with the strong correlation between the various activities and the extract components determined through HPLC–PDA.

## Conclusion

This study concludes that *Monotheca buxifolia* contains a diverse range of phytochemicals with significant biological activities. The stem extract contained a high amount of phenolics, while flavonoids were most prominent in the leaf extract. The free-radical-scavenging activity, reducing power, and antioxidative potential show that the plant can be used as a nutraceutical to heal stress in the body. Furthermore, the significant enzyme-inhibitory activity, especially against AChE, BChE, and tyrosinase, shows that the plant is clinically important as a potential cure for diseases. The prominent amylase- and glycosidase-inhibitory activity of the extracts is in accordance with folk knowledge linking this plant with digestive and laxative properties. The UHPLC–MS analysis and HPLC–PDA quantification identified a number of bioactive secondary metabolites, including phenolics, flavonoids, alkaloids, and terpenoid derivatives, which may be responsible for the plant’s significant antioxidant and enzyme-inhibitory potential. The observed activities of this plant could act as a starting point in the identification and isolation of bioactive compounds with antioxidant and enzyme-inhibitory potential.

## Data Availability

All the data of this study is included in the manuscript.

## Abbreviations

ABTS: 2,2′-Azino-bis(3-ethylbenzothiazoline-6-sulfonic acid) diammonium salt
ACAE: acarbose equivalent
AChE: acetylcholinesterase
BChE: butyrylcholinesterase
CUPRAC: CUPric Reducing Antioxidant Capacity
DPPH: 2,2-diphenyl-1-picrylhydrazyl
EDTAE: EDTA equivalent
FRAP: Ferric-reducing antioxidant power
GAE: gallic acid equivalent
GALAE: galantamine equivalents
HPLC-PDA: high performance liquid chromatography equipped with photodiode array detector
KAE: kojic acid equivalent
PCA: Principal Component Analysis
QE: quercetin equivalent
RE: rutin equivalents
TE: Trolox equivalent
UHPLC-MS: Ultrahigh performance liquid chromatography-mass spectrometer
